# Sodium-glucose co-transporter 2 inhibitors and new-onset diabetes in cardiovascular or kidney disease

**DOI:** 10.1093/eurheartj/ehae780

**Published:** 2024-11-21

**Authors:** John W Ostrominski, Mats C Højbjerg Lassen, Brian L Claggett, Zi Michael Miao, Silvio E Inzucchi, Kieran F Docherty, Akshay S Desai, Pardeep S Jhund, Lars Køber, Piotr Ponikowski, Marc S Sabatine, Carolyn S P Lam, Felipe A Martinez, Rudolf A de Boer, Adrian F Hernandez, Sanjiv J Shah, Magnus Petersson, Anna Maria Langkilde, John J V McMurray, Scott D Solomon, Muthiah Vaduganathan

**Affiliations:** Cardiovascular Division, Brigham and Women’s Hospital, Harvard Medical School, 75 Francis St., Boston, MA 02115, USA; Division of Endocrinology, Diabetes and Hypertension, Brigham and Women’s Hospital, Harvard Medical School, Boston, MA, USA; Cardiovascular Division, Brigham and Women’s Hospital, Harvard Medical School, 75 Francis St., Boston, MA 02115, USA; Department of Cardiology, Copenhagen University Hospital—Herlev and Gentofte, Copenhagen, Denmark; Center for Translational Cardiology and Pragmatic Randomized Trials, Department of Biomedical Sciences, Faculty of Health and Medical Sciences, University of Copenhagen, Denmark; Cardiovascular Division, Brigham and Women’s Hospital, Harvard Medical School, 75 Francis St., Boston, MA 02115, USA; Cardiovascular Division, Brigham and Women’s Hospital, Harvard Medical School, 75 Francis St., Boston, MA 02115, USA; Section of Endocrinology, Yale University School of Medicine, New Haven, CT, USA; British Heart Foundation Cardiovascular Research Centre, University of Glasgow, Glasgow, UK; Cardiovascular Division, Brigham and Women’s Hospital, Harvard Medical School, 75 Francis St., Boston, MA 02115, USA; British Heart Foundation Cardiovascular Research Centre, University of Glasgow, Glasgow, UK; Rigshospitalet, Copenhagen University Hospital, Copenhagen, Denmark; Wroclaw Medical University, Wroclaw, Poland; TIMI Study Group, Cardiovascular Division, Brigham and Women’s Hospital, Harvard Medical School, Boston, MA, USA; National Heart Centre Singapore and Duke-National University of Singapore, Singapore; Universidad Nacional de Córdoba, Córdoba, Argentina; Department of Cardiology, Erasmus Medical Center, Rotterdam, The Netherlands; Division of Cardiology, Duke University School of Medicine, Durham, NC, USA; Duke Clinical Research Institute, Duke University, Durham, NC, USA; Feinberg Cardiovascular Research Institute, Northwestern University Feinberg School of Medicine, Chicago, IL, USA; Late-Stage Development, Cardiovascular, Renal, and Metabolism, BioPharmaceuticals R&D, AstraZeneca, Gothenburg, Sweden; Late-Stage Development, Cardiovascular, Renal, and Metabolism, BioPharmaceuticals R&D, AstraZeneca, Gothenburg, Sweden; British Heart Foundation Cardiovascular Research Centre, University of Glasgow, Glasgow, UK; Cardiovascular Division, Brigham and Women’s Hospital, Harvard Medical School, 75 Francis St., Boston, MA 02115, USA; Cardiovascular Division, Brigham and Women’s Hospital, Harvard Medical School, 75 Francis St., Boston, MA 02115, USA

**Keywords:** Heart failure, Diabetes, Sodium-glucose co-transporter 2 inhibitors, Prevention

## Abstract

**Background and Aims:**

Individuals with heart failure (HF), other forms of cardiovascular disease, or kidney disease are at increased risk for the development and adverse health effects of diabetes. As such, prevention or delay of diabetes is an important treatment priority in these groups. The aim of this meta-analysis was to determine the effect of sodium-glucose co-transporter 2 inhibitors (SGLT2i) on incident diabetes in HF across the spectrum of left ventricular ejection fraction (LVEF) and across the broader spectrum of cardiovascular or kidney disease.

**Methods:**

First, the effects of dapagliflozin vs. placebo on new-onset diabetes were assessed in a pooled, participant-level analysis of the DAPA-HF and DELIVER trials. New-onset diabetes was defined as the new initiation of glucose-lowering therapy during follow-up, and time from randomization to new-onset diabetes was evaluated using Cox proportional hazards models. Second, PubMed and Embase were searched to identify large-scale randomized clinical outcomes trials (RCTs) comparing SGLT2i with placebo among adults with cardiovascular or kidney disease. A trial-level meta-analysis was then conducted to summarize the treatment effects of SGLT2i on the incidence of new-onset diabetes.

**Results:**

In the pooled analysis of DAPA-HF and DELIVER including 5623 participants with HF but without diabetes at baseline, dapagliflozin reduced the incidence of new-onset diabetes by 33% [hazard ratio (HR), 0.67; 95% confidence interval (CI), .49–.91; *P* = .012] when compared with placebo. There was no evidence of heterogeneity across the spectrum of continuous LVEF or key subgroups. Among seven complementary RCTs including 17 855 participants with cardiovascular or kidney disease, SGLT2i reduced the of new-onset diabetes by 26% (HR, 0.74; 95% CI .65–.85; *P* < .001), with consistent effects across trials.

**Conclusions:**

SGLT2i reduced the incidence of new-onset diabetes among individuals with cardiovascular or kidney disease. These findings suggest that SGLT2i implementation may have an important ancillary benefit on prevention or delay of diabetes in these high-risk populations.

## Structured Graphical Abstract

**Structured Graphical Abstract:**
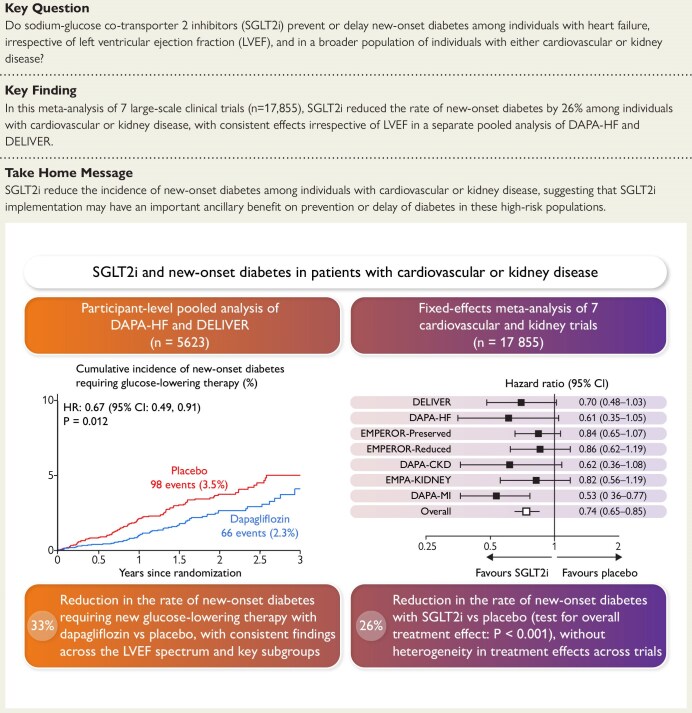
Findings of a participant-level pooled analysis of the DAPA-HF and DELIVER trials evaluating treatment effects of dapagliflozin vs. placebo on new-onset diabetes requiring initiation of glucose-lowering therapy (left). Findings of a fixed-effects meta-analysis of seven complementary cardiovascular and kidney trials evaluating the effect of SGLT2i on new-onset diabetes (right). DAPA-CKD, Dapagliflozin and Prevention of Adverse Outcomes in Chronic Kidney Disease; DAPA-HF, Dapagliflozin and Prevention of Adverse Outcomes in Heart Failure; DAPA-MI, Dapagliflozin in Patients with Myocardial Infarction; DELIVER, Dapagliflozin Evaluation to Improve the Lives of Patients with Preserved Ejection Fraction Heart Failure; EMPA-KIDNEY, Study of Heart and Kidney Protection with Empagliflozin; EMPEROR-Preserved, Empagliflozin Outcome Trial in Patients with Chronic Heart Failure with Preserved Ejection Fraction; EMPEROR-Reduced, Empagliflozin Outcome Trial in Patients with Chronic Heart Failure and a Reduced Ejection Fraction; HR, hazard ratio; LVEF, left ventricular ejection fraction; SGLT2i, sodium-glucose co-transporter 2 inhibitor.

**See the editorial comment for this article ‘The recent renewed interest among cardiologists in detecting and preventing diabetes is welcomed’, by A. Norhammar and V. Ritsinger, https://doi.org/10.1093/eurheartj/ehae833.**

## Introduction

Insulin resistance and other forms of metabolic impairment are central to heart failure (HF) pathophysiology.^1,2^ As such, individuals with HF have a higher risk of developing diabetes when compared to the general population.^1,3,4^ Once established, concurrent HF and diabetes are associated with an additive risk of death, disability, multimorbidity, polypharmacy, and excess healthcare expenditures.^5^ Prevention or delay of diabetes, therefore, is an important priority of comprehensive efforts aiming to improve health outcomes in this high-risk population.

Sodium-glucose co-transporter 2 inhibitors (SGLT2i), originally developed as glucose-lowering therapies (GLTs), are presently indicated as a foundational component of comprehensive pharmacotherapy in HF, irrespective of left ventricular ejection fraction (LVEF) and glycaemic status.^6,7^ Despite minimal impact on glycated haemoglobin among individuals with either normoglycaemia or mild dysglycaemia,^8,9^ SGLT2i have salutary metabolic effects of relevance for insulin sensitivity and diabetes risk.^10^ Therefore, as the majority of patients with HF does not have diabetes,^1^ optimal SGLT2i implementation in this population provides substantial additional opportunity for diabetes prevention.

Beyond LVEF, HF with reduced ejection fraction (HFrEF) and HF with preserved ejection fraction (HFpEF) represent pathophysiologically distinct entities with potentially unique mechanisms of metabolic impairment. Namely, insulin resistance in HFrEF may be driven by sarcopenia,^11^ while excess/dysfunctional adiposity, impaired skeletal muscle energetics,^12^ and systemic inflammation may be more dominant factors in HFpEF.^13^ SGLT2i have previously been shown to reduce the rate of new-onset diabetes among individuals with HFrEF,^8,9^ but whether these benefits apply across the diverse HF spectrum remains unknown.

Herein, we first conducted a participant-level pooled analysis of the DAPA-HF (Dapagliflozin and Prevention of Adverse Outcomes in Heart Failure) and DELIVER (Dapagliflozin Evaluation to Improve the Lives of Patients with Preserved Ejection Fraction Heart Failure) trials to evaluate the effects of dapagliflozin on new-onset diabetes in HF across the spectrum of LVEF. As insulin resistance and diabetes are also common and important therapeutic targets in individuals with kidney disease,^14^ we then carried out a broader trial-level meta-analysis of seven complementary cardiovascular and kidney disease outcomes trials to summarize the effects of SGLT2i on new-onset diabetes.

## Methods

### Study design and participants

This analysis combines data from two international, multicentre, phase 3, randomized, double-blind, placebo-controlled, clinical trials: DAPA-HF (NCT03036124) and DELIVER (NCT03619213). Details of the trials’ design and study protocols have been previously published.^15,16^

The DAPA-HF trial was performed across 410 sites in 20 countries (Argentina, Brazil, Bulgaria, Canada, China, Czech Republic, Denmark, Germany, Hungary, India, Japan, The Netherlands, Poland, Russia, Slovakia, Sweden, Taiwan, UK, USA, and Vietnam).^15^ The primary objective was to determine whether dapagliflozin reduced the incidence of worsening HF or cardiovascular death among individuals with HFrEF. Major inclusion criteria included New York Heart Association (NYHA) functional class II–IV symptoms, LVEF ≤ 40%, and elevated serum N-terminal pro-B-type natriuretic peptide (NT-proBNP) concentrations. Key exclusion criteria were a history of type 1 diabetes and an estimated glomerular filtration rate (eGFR) < 30 mL/min/1.73 m^2^.

The DELIVER trial was performed across 353 sites in 20 countries (Argentina, Belgium, Brazil, Bulgaria, Canada, China, Czech Republic, Hungary, Japan, Mexico, The Netherlands, Peru, Poland, Romania, Russia, Saudi Arabia, Spain, Taiwan, USA, and Vietnam).^16^ The primary objective was to determine whether dapagliflozin reduced the incidence of worsening HF or cardiovascular death among individuals with HF and mildly reduced or preserved ejection fraction. Major inclusion criteria included NYHA functional class II–IV symptoms, LVEF > 40%, evidence of structural heart disease, and elevated serum NT-proBNP concentrations. Key exclusion criteria were a history of type 1 diabetes and eGFR < 25 mL/min/1.73 m^2^.

All participants in both trials provided written informed consent. The trials were approved by the ethics committee at each centre and were conducted in accordance with the International Conference on Harmonization Good Clinical Practice guideline and the Declaration of Helsinki.

In this pooled, participant-level analysis of the DAPA-HF and DELIVER trials, we evaluated the effects of dapagliflozin vs. placebo on new-onset diabetes. Participants with no history of diabetes, baseline glycated haemoglobin (HbA1c) < 6.5%, and not using GLTs at baseline were included in this analysis. Included participants were then classified as having pre-diabetes and normoglycaemia at baseline according to the American Diabetes Association definitions of HbA1c 5.7%–6.4% and <5.7%, respectively.^17^

### Randomization and masking

Participants were randomly assigned (1:1) to receive dapagliflozin or placebo in both DAPA-HF and DELIVER. Randomization was stratified according to diabetes status in both trials. Participants and all trial personnel were masked to group assignment in both studies. Detailed overviews of the randomization and masking procedures employed in the DAPA-HF and DELIVER trials have been published previously.^15,16^

### Procedures

Participants assigned to the dapagliflozin arms received 10 mg orally once daily, with matching placebo in the placebo combined with standard care. In DAPA-HF, post-randomization in-person follow-up visits were conducted 14 days, then 2, 4, and 8 months, and then continued at 4-month intervals thereafter. In DELIVER, post-randomization in-person follow-up visits were conducted at 30 days, then 4 and 8 months, and then continued at 4-month intervals thereafter. All participants underwent HbA1c testing in the non-fasting state at baseline through a central laboratory, using the Bio-Rad VARIANT II ion-exchange high-performance liquid chromatography assay (Bio-Rad Laboratories, Hercules, CA, USA).

### Outcomes

As serial HbA1c testing was not performed in DELIVER, the incidence of new-onset diabetes in this pooled analysis was defined as new initiation of a GLT during trial follow-up.

### Meta-analysis search strategy and selection criteria

To examine the totality of evidence, we conducted a meta-analysis with inverse-variance weighting to assess the composite effects of SGLT2i vs. placebo on new-onset diabetes across trials of HF [DELIVER, DAPA-HF, EMPEROR-Preserved (Empagliflozin Outcome Trial in Patients with Chronic Heart Failure with Preserved Ejection Fraction), and EMPEROR-Reduced (Empagliflozin Outcome Trial in Patients with Chronic Heart Failure and a Reduced Ejection Fraction)],^15,16,18–20^ chronic kidney disease [DAPA-CKD (Dapagliflozin and Prevention of Adverse Outcomes in Chronic Kidney Disease) and EMPA-KIDNEY (Study of Heart and Kidney Protection with Empagliflozin)],^21,22^ and after acute myocardial infarction [DAPA-MI (Dapagliflozin in Patients with Myocardial Infarction)].^23^ To ensure important trials were not inadvertently omitted, we performed a systematic review of the literature via PubMed and Embase to identify randomized, placebo-controlled, trials including participants with cardiovascular or kidney disease published between 1 January 2015 and 1 January 2024. To capture trials designed to evaluate clinical outcomes, we limited our selection to trials enrolling ≥1000 participants.^24^ Trials including only individuals with diabetes were excluded. The pre-registered search query, performed on 15 January 2024, is detailed in Supplementary Methods. Key design features and definitions of new-onset diabetes in each trial are summarized in Supplementary data online, *Table S1*. No additional trials were identified that met criteria for inclusion (see Supplementary data online, *Figure S1*). Data were extracted using standardized forms for outcomes of interest by two authors (J.W.O. and M.C.H.L.), and any discrepancies were resolved by consensus.

### Statistical analysis

Characteristics were compared between participants with and without new-onset diabetes using a two-sample *t*-test and χ^2^ test for continuous and categorical variables, respectively. The effect of dapagliflozin vs. placebo on new-onset diabetes was evaluated using Kaplan–Meier estimates and hazard ratios (HRs), with 95% confidence intervals (CIs) derived from Cox proportional hazards regression models stratified by study. The heterogeneity of treatment effect between studies was assessed via inclusion of an interaction term between treatment and study. Consistency of treatment effects across key subgroups [age, sex, race, region, LVEF category, and glycaemic status (pre-diabetes and normoglycaemia)] was additionally explored using Cox proportional hazards regression models containing an additional interaction term between treatment and each subgroup. We additionally evaluated the effects on new-onset diabetes after adjusting for baseline HbA1c. The effect of dapagliflozin on new-onset diabetes according to baseline LVEF, body mass index, and HbA1c as continuous variables was additionally modelled using restricted cubic splines, with the number of knots selected to minimize the Akaike information criterion. As some GLTs are indicated for conditions other than diabetes (e.g. pre-diabetes and overweight/obesity), we conducted a sensitivity analysis considering only new initiation of a GLT other than metformin or glucagon-like peptide-1 receptor agonists.

To examine the totality of evidence, we additionally conducted a fixed-effects meta-analysis with inverse-variance weighting to assess the composite effects of SGLT2i vs. placebo on new-onset diabetes across large-scale, randomized, placebo-controlled, outcomes trials of HF, chronic kidney disease, and after acute myocardial infarction. Between-trial heterogeneity of treatment effect on new-onset diabetes, as defined in each trial, was evaluated using Cochran’s Q test.

All analyses were conducted using Stata version 17.0 (StataCorp; College Station, TX, USA), and two-sided *P*-values of <.05 were considered statistically significant.

### Role of funding source

The sponsor (AstraZeneca) of the study was not involved in the design of the present study, the analysis, interpretation of data, writing of the report, or ultimate decision to submit the paper for publication. Both the DAPA-HF and DELIVER trials were sponsored by AstraZeneca as a collaboration between the sponsor and academic-led steering committees.

## Results

### Participant-level meta-analysis of DAPA-HF and DELIVER

In a pooled analysis of DAPA-HF and DELIVER, 5623 participants without diabetes or GLT use at baseline [*n* = 2530 (45%) enrolled in DAPA-HF and *n* = 3093 (55%) enrolled in DELIVER] were included. Over a median follow-up of 22 months, 164 (2.9%) developed new-onset diabetes requiring GLT (incidence rate 1.58; 95% CI 1.35–1.84 per 100 person-years), 121 (74%) of whom had pre-diabetes at baseline. The incidence rate of new-onset diabetes was similar (incidence rate 1.51; 95% CI 1.16–1.96 per 100 person-years) in DAPA-HF compared with DELIVER (incidence rate 1.61; 95% CI 1.34–1.95 per 100 person-years). As compared to those who did not develop diabetes during trial follow-up, participants with new-onset diabetes were more likely to be female and be Asian or Black/African American, have higher body mass index and baseline HbA1c, and lower baseline health-related quality of life and kidney function (*Table 1*).

**Table 1:** Baseline characteristics for participants with and without new-onset diabetes in a pooled analysis of DAPA-HF and DELIVER

Characteristic	No new-onset diabetes (*n* = 5459)	New-onset diabetes (*n* = 164)	*P*-value
Age, years	69.6 ± 11.2	69.1 ± 10.4	.55
Men	3511 (64.3%)	87 (53.0%)	.003
Randomized to dapagliflozin	2744 (50.3%)	66 (40.2%)	.011
Region			.028
Europe and Saudi Arabia	2560 (46.9%)	60 (36.6%)	
North America	734 (13.4%)	29 (17.7%)	
South America	941 (17.2%)	27 (16.5%)	
Asia/Pacific	1224 (22.4%)	48 (29.3%)	
Race^a^			<.001
American Indian or Alaska Native	88 (1.6%)	1 (.6%)	
Asian	1245 (22.8%)	48 (29.3%)	
Black or African American	149 (2.7%)	9 (5.5%)	
Native Hawaiian or Other Pacific Islander	0 (.0%)	1 (.6%)	
White	3835 (70.3%)	103 (62.8%)	
Other	142 (2.6%)	2 (1.2%)	
Baseline LVEF, %	43.7 ± 14.2	45.8 ± 13.8	.06
Baseline pulse, beats/min	70.8 ± 11.7	70.7 ± 12.4	.96
Baseline systolic blood pressure, mmHg	124.1 ± 15.9	124.0 ± 15.9	.94
Baseline diastolic blood pressure, mmHg	73.8 ± 10.4	72.8 ± 9.7	.21
Baseline body mass index, kg/m^2^	28.0 ± 5.8	30.0 ± 7.1	<.001
History of hypertension	4187 (76.7%)	133 (81.1%)	.19
Prior HF hospitalization	2306 (42.2%)	67 (40.9%)	.72
Trial			.003
Enrolled in DAPA-HF	2475 (45.3%)	55 (33.5%)	
Enrolled in DELIVER	2984 (54.7%)	109 (66.5%)	
NYHA functional class^b^			.52
II	4067 (74.5%)	116 (70.7%)	
III	1365 (25.0%)	48 (29.3%)	
IV	26 (.5%)	0 (.0%)	
Baseline KCCQ-Total Symptom Score	73.5 ± 21.1	66.8 ± 25.9	<.001
Baseline eGFR, mL/min/1.73 m^2^	65.0 ± 19.1	60.2 ± 19.0	.002
Baseline eGFR < 60 mL/min/1.73 m^2^	2235 (40.9%)	84 (51.2%)	.008
Baseline HbA1c, %	5.7 ± .4	5.9 ± .4	<.001
Baseline HbA1c ≥ 5.7%–<6.5%	3328 (61.0%)	121 (73.8%)	<.001
Baseline creatinine, μmol/L	99.4 ± 28.1	106.2 ± 32.4	.003
Loop diuretic	4124 (75.5%)	136 (82.9%)	.030
ACEi	2516 (46.1%)	63 (38.4%)	.05
ARB	1632 (29.9%)	59 (36.0%)	.09
ACEi/ARB	4130 (75.7%)	122 (74.4%)	.71
ARNi	426 (7.8%)	12 (7.3%)	.82
Beta-blocker	4795 (87.8%)	145 (88.4%)	.82
MRA	3076 (56.3%)	96 (58.5%)	.58
Statin	3199 (58.6%)	107 (65.2%)	.09
Antiplatelet	2284 (41.8%)	72 (43.9%)	.60
Anticoagulant	2820 (51.7%)	83 (50.6%)	.79
CRT-D or ICD	554 (10.1%)	19 (11.6%)	.55

Values are mean ± SD or *n* (%). *P*-values computed via two-sample *t*-test and χ^2^ testing for continuous and categorical variables, respectively.

ACEi, angiotensin-converting enzyme inhibitor; ARB, angiotensin receptor blocker; ARNi, angiotensin receptor-neprilysin inhibitor; CRT-D, cardiac resynchronization therapy defibrillator; eGFR, estimated glomerular filtration rate; HbA1c, glycated haemoglobin; HF, heart failure; ICD, implantable cardioverter-defibrillator; KCCQ, Kansas City Cardiomyopathy Questionnaire; LVEF, left ventricular ejection fraction; MRA, mineralocorticoid receptor antagonist; NYHA, New York Heart Association.

^a^Captured on a dedicated demographics case report form, which included the following categories: Asian, Black or African American, White, or other race designation (including Native Hawaiian or Other Pacific Islander, American Indian, or Alaska Native).

^b^One participant in the placebo group of the DELIVER trial, and who did not develop new-onset diabetes, had NYHA functional class I at baseline and was not included in the analysis of this variable.

**Figure 1:**
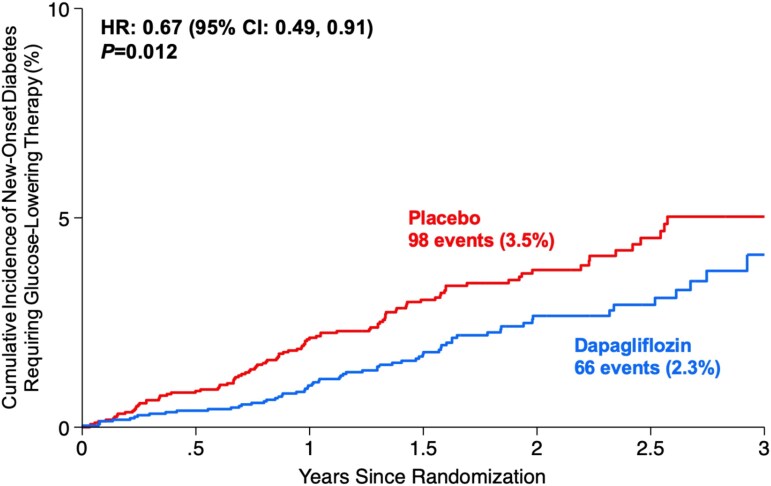
Cumulative incidence of new-onset diabetes by assigned treatment in a pooled analysis of DAPA-HF and DELIVER. HR, hazard ratio

Overall, 98 (3.5%) participants in the placebo arm developed new-onset diabetes requiring GLT (incidence rate 1.89; 95% CI 1.55–2.30 per 100 person-years), compared with 66 (2.3%) in the dapagliflozin arm (incidence rate 1.26; 95% CI .99–1.61 per 100 person-years). Dapagliflozin significantly reduced the rate of incident diabetes (HR 0.67; 95% CI .49–.91; *P* = .012) when compared with placebo (*Figure 1*), with an estimated number needed to treat (NNT) of 83 individuals to prevent one occurrence of incident diabetes requiring GLT over trial follow-up. Treatment effects of dapagliflozin on incident diabetes were consistent between the clinical trials (*P*_interaction_ = .67) (see Supplementary data online, *Figure S2*) and between participants with normoglycaemia (HR 0.83; 95% CI .46–1.05) and pre-diabetes (HR 0.62; 95% CI .43–.90) at baseline (*P*_interaction_ = .43). Similar findings were observed after adjustment for baseline HbA1c (HR 0.67; 95% CI .49–.92; *P* = .012), and when only considering new initiation of GLT other than metformin or glucagon-like peptide-1 receptor agonists (HR 0.66; 95% CI .45–.98; *P* = .04) in a sensitivity analysis.

**Figure 2:**
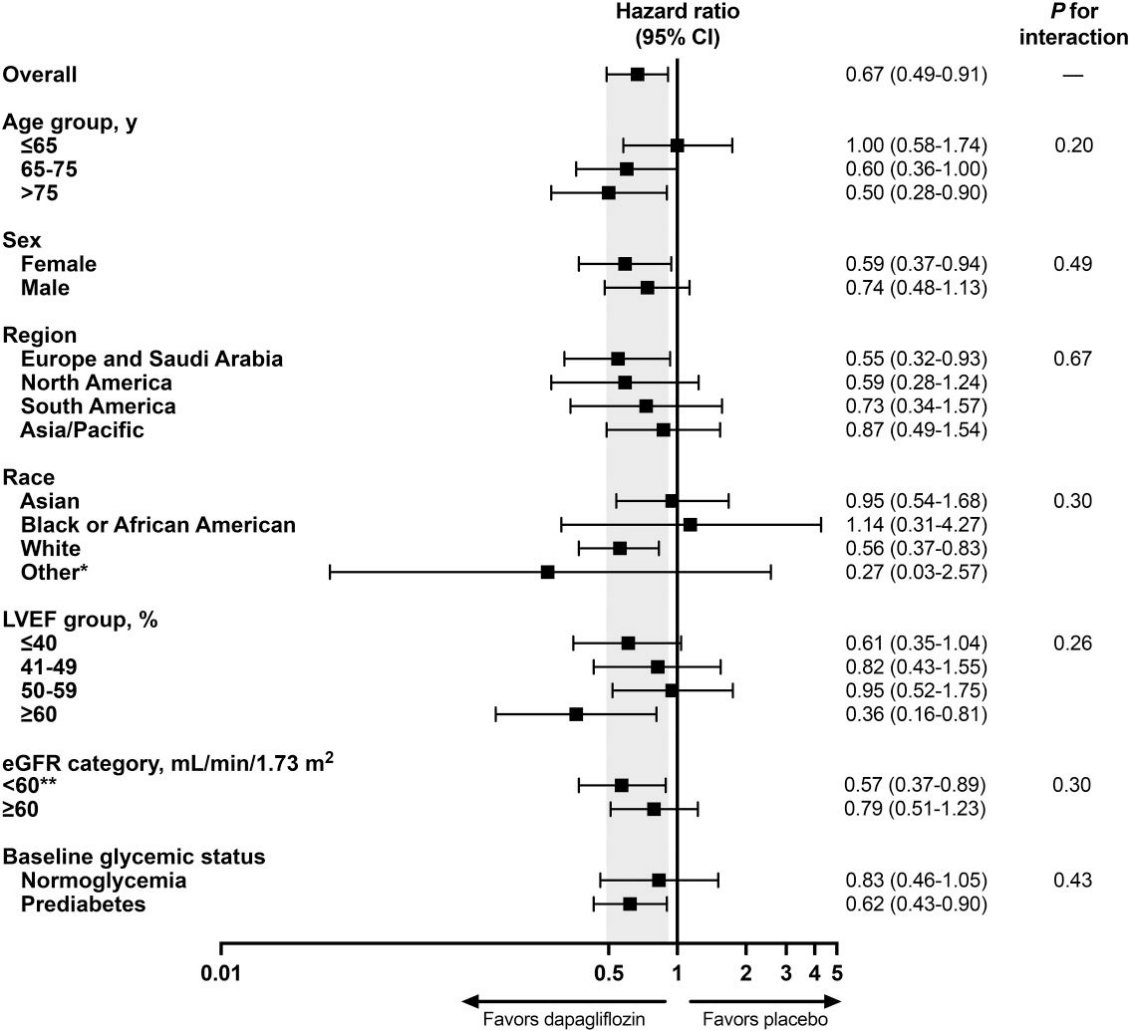
Treatment effects of dapagliflozin on new-onset diabetes in a pooled analysis of DAPA-HF and DELIVER across key subgroups. *Other race designation as captured on demographics case report forms (including Native Hawaiian or Other Pacific Islander, American Indian, or Alaska Native). **eGFR < 30 mL/min/1.73 m^2^ and <25 mL/min/1.73 m^2^ were exclusionary in DAPA-HF and DELIVER, respectively. Abbreviations as in *Table 1*

**Figure 3:**
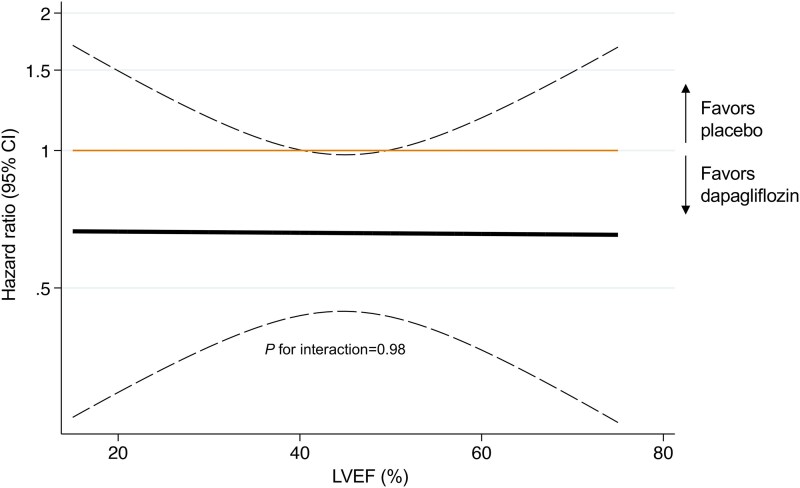
Effect of dapagliflozin vs. placebo on new-onset diabetes in a pooled analysis of DAPA-HF and DELIVER across LVEF assessed as a continuous measure. Estimated hazard ratios (solid black line) and 95% confidence intervals (dotted black lines) obtained from Cox proportional hazards models with continuous left ventricular ejection fraction and expressed via a linear model. Abbreviations as in *Table 1*

Benefits of dapagliflozin were additionally consistent across age, sex, race, LVEF category, baseline glycaemic status, and eGFR category (*Figure 2*). When LVEF was modelled continuously, there was no evidence of heterogeneity (*P*_interaction_ = .98) in the treatment effects of dapagliflozin vs. placebo on new-onset diabetes across the LVEF spectrum (*Figure 3*). Similar findings were observed when treatment effects of dapagliflozin was evaluated across continuous baseline body mass index (*P*_interaction_ = .62) and HbA1c (*P*_interaction_ = .89) (see Supplementary data online, *Figure S3*).

Irrespective of treatment assignment, the incidence of serious adverse safety events was higher among participants who developed new-onset diabetes as compared to those who did not, but no major hypoglycaemia events were observed in either subgroup (i.e. all participants without diabetes and no GLT use at baseline). Safety events were well-balanced between treatment arms (*Table 2*).

**Table 2:** Safety events overall and between treatment arms, by new-onset diabetes status in a pooled analysis of DAPA-HF and DELIVER

	No new-onset diabetes			New-onset diabetes			
Event	All (*n* = 5459)	Dapagliflozin (*n* = 2744)	Placebo (*n* = 2715)	All (*n* = 164)	Dapagliflozin (*n* = 66)	Placebo (*n* = 98)	*P* for interaction^a^
Any serious adverse event	2021 (37.1%)	1003 (36.6%)	1018 (37.5%)	117 (71.3%)	45 (68.2%)	72 (73.5%)	.54
Any adverse event leading to drug discontinuation	292 (5.4%)	157 (5.7%)	135 (5.0%)	15 (9.1%)	6 (9.1%)	9 (9.2%)	.78
Any adverse event leading to dose interruption	685 (12.6%)	314 (11.5%)	371 (13.7%)	48 (29.3%)	18 (27.3%)	30 (30.6%)	.92
Any definite or probable ketoacidosis	0 (0.0%)	0 (0.0%)	0 (0.0%)	0 (0.0%)	0 (0.0%)	0 (0.0%)	
Any major hypoglycaemic event	0 (0.0%)	0 (0.0%)	0 (0.0%)	0 (0.0%)	0 (0.0%)	0 (0.0%)	

^a^Evaluating effect modification of the incidence of each safety event by assigned treatment.

### Trial-level meta-analysis of cardiovascular and kidney outcomes trials

Overall, 35 655 participants (mean age, 67 years; 33% female) were enrolled in the DELIVER, DAPA-HF, EMPEROR-Preserved, EMPEROR-Reduced, DAPA-CKD, EMPA-KIDNEY, and DAPA-MI trials. Of these, 17 855 were evaluated in secondary analyses evaluating treatment effects of SGLT2i on new-onset diabetes and included in the trial-level meta-analysis. Key baseline characteristics of participants in each full trial population are summarized in Supplementary data online, *Table S2*.

**Figure 4:**
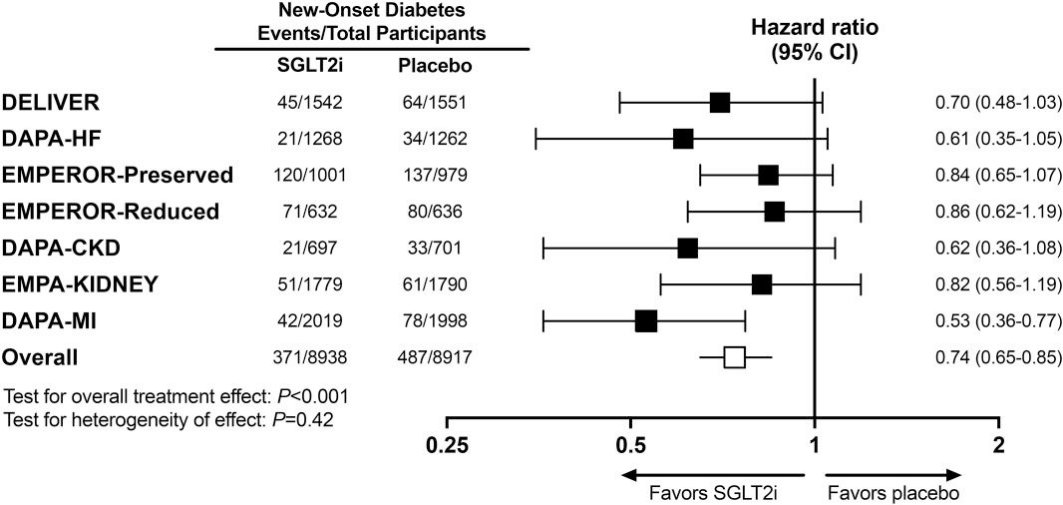
Treatment effects of SGLT2i vs. placebo on new-onset diabetes across trials of heart failure, chronic kidney disease, and myocardial infarction. The definition of new-onset diabetes varied across the trials included in this meta-analysis. Treatment effect estimates and 95% CIs for DELIVER and DAPA-HF reflect the definition for new-onset diabetes employed in this analysis, namely new introduction of glucose-lowering therapy. Serial HbA1c was available in EMPEROR-Preserved, EMPEROR-Reduced, DAPA-CKD, EMPA-KIDNEY, and DAPA-MI and was used as part of the definition of new-onset diabetes in these trials. In EMPEROR-Preserved and EMPEROR-Reduced, new-onset diabetes was evaluated only among participants with pre-diabetes at baseline and was defined as ≥1 HbA1c ≥ 6.5% or an investigator diagnosis. In DAPA-CKD, new-onset diabetes was defined as a ≥1 HbA1c ≥ 6.5%. In EMPA-KIDNEY, new-onset diabetes was defined as a clinical diagnosis, initiation of glucose-lowering therapy, or ≥1 HbA1c ≥ 6.5%. In DAPA-MI, new-onset diabetes was defined as an investigator diagnosis requiring glucose-lowering therapy or HbA1c ≥ 6.5% at two consecutive time points. DAPA-CKD, Dapagliflozin and Prevention of Adverse Outcomes in Chronic Kidney Disease; DAPA-HF, Dapagliflozin and Prevention of Adverse Outcomes in Heart Failure; DAPA-MI, Dapagliflozin in Patients with Myocardial Infarction; DELIVER, Dapagliflozin Evaluation to Improve the Lives of Patients with Preserved Ejection Fraction Heart Failure; EMPA-KIDNEY, Study of Heart and Kidney Protection with Empagliflozin; EMPEROR-Preserved, Empagliflozin Outcome Trial in Patients with Chronic Heart Failure with Preserved Ejection Fraction; EMPEROR-Reduced, Empagliflozin Outcome Trial in Patients with Chronic Heart Failure and a Reduced Ejection Fraction; HbA1c, glycated haemoglobin; SGLT2i, sodium-glucose co-transporter 2 inhibitor

Of 8938 participants randomized to treatment with SGLT2i, 371 (4.2%) developed new-onset diabetes, compared with 487 of 8917 (5.5%) participants randomized to placebo over trial follow-up. Treatment with either dapagliflozin or empagliflozin was associated with a 26% lower rate of new-onset diabetes (HR 0.74; 95% CI .65–.85; *P* < .001) (*Figure 4*), with an estimated NNT of 77 individuals to prevent one occurrence of incident diabetes over cumulative trial follow-up (range of median follow-up in included trials, 11.6 months to 2.4 years).

No heterogeneity in the treatment effects of SGLT2i was observed across the seven trials (*P* for test of heterogeneity = .42). Similar findings were observed in HF trials (DAPA-HF, EMPEROR-Reduced, DELIVER, and EMPEROR-Preserved; HR 0.79; 95% CI .67–.94) compared with non-HF trials (DAPA-MI, DAPA-CKD, and EMPA-KIDNEY; HR 0.65; 95% CI .51–.83) (*P* for test of heterogeneity = .20). However, while the rate of new-onset diabetes was lower compared with placebo in trials evaluating both dapagliflozin (HR 0.61; 95% CI .49–.76) and empagliflozin (HR 0.84; 95% CI .71–1.00), heterogeneity was observed favouring greater benefits in trials evaluating dapagliflozin (*P* for test of heterogeneity = .026).

## Discussion

In this participant-level pooled analysis of DELIVER and DAPA-HF, we show that dapagliflozin consistently reduced the rate of new-onset diabetes necessitating the initiation of GLT among individuals with HF, irrespective of age, sex, race, baseline HbA1c, and LVEF, without excess risk of hypoglycaemia. While most new-onset diabetes events occurred among individuals with pre-diabetes, we did not identify heterogeneity in treatment effects of dapagliflozin by baseline glycaemic status. Additionally, in a meta-analysis integrating new estimates from participant-level data from DELIVER and DAPA-HF with published trial-level estimates from five contemporary cardiovascular-kidney trials, we estimate a 26% reduction in new-onset diabetes with SGLT2i vs. placebo. To our knowledge, this comprehensive meta-analysis spanning nearly 18 000 participants examines the totality of evidence from all cardiovascular and kidney outcome trials of SGLT2i that have been conducted inclusive of individuals without diabetes. Overall, these findings extend understanding of the wide-ranging cardiovascular-kidney-metabolic benefits of SGLT2i, highlighting the potential of robust implementation efforts to reduce the burden of diabetes across the HF spectrum (*Structured Graphical Abstract*).

Due, in part, to population aging and an expanding prevalence of shared risk factors (e.g. overweight and obesity), the prevalence of concurrent diabetes and cardiovascular disease, including HF, is substantial and growing in the general population.^25^ Bidirectional aetiopathogenic interactions between diabetes and HF also contribute to these trends. Although diabetes is a well-established risk factor for HF onset and progression, HF as a risk factor for incident diabetes is less widely appreciated despite the high prevalence of insulin resistance and pre-diabetes in this population.^1,5^ Among individuals with diabetes in UKPDS (UK Prospective Diabetes Study), the incidence of new-onset HF was 11.9 per 1000 patient-years, which is similar to findings from major SGLT2i cardiovascular outcome trials and population-based observational studies.^5,26^ Alternatively, in the CHARM (Candesartan in Heart Failure Assessment of Reduction in Mortality and Morbidity) Program, which included participants with chronic HF across the LVEF spectrum, the incidence of new-onset diabetes was 27.8 per 1000 patient-years.^27^ As such, diabetes commonly occurs after HF onset,^3,4^ stressing the importance of rigorous screening and prevention efforts in this population.

Lifestyle interventions and multiple oral GLTs (i.e. metformin, thiazolidinediones, and α-glucosidase inhibitors) have been shown to reduce incident diabetes,^28^ but no prospective diabetes prevention studies have evaluated SGLT2i or targeted populations with HF or CKD. Diabetes prevention in HF and CKD, therefore, remains an important clinical and research gap. However, SGLT2i have demonstrated important benefits on incident diabetes in completed cardiovascular and kidney disease outcomes trials. In a pooled analysis of DAPA-HF and DAPA-CKD, dapagliflozin was associated with a 33% reduction in new-onset diabetes compared with placebo.^8^ In DAPA-MI, which enrolled individuals with acute myocardial infarction and left ventricular systolic dysfunction but without HF or diabetes, dapagliflozin was associated with a 47% reduction in the rate of new-onset diabetes.^23^ Empagliflozin was additionally associated with a 14% lower rate of a pre-specified secondary endpoint of new-onset diabetes in the EMPEROR-Reduced trial,^18^ but was assessed only in participants with pre-diabetes at baseline and did not reach statistical significance. Similar findings were observed in the EMPEROR-Preserved and EMPA-KIDNEY trials.^19,22^ While both dapagliflozin and empagliflozin reduced new-onset diabetes in this comprehensive meta-analysis, modest heterogeneity was observed favouring greater reduction in new-onset diabetes in trials evaluating dapagliflozin compared with empagliflozin. This observation may relate to variation in the characteristics of the respective trial populations, in definitions of new-onset diabetes, or, less likely, in study drug pharmacodynamics. The findings presented herein support and extend the totality of these data, showing that benefits of SGLT2i on new-onset diabetes extend across the cardiovascular-kidney-metabolic spectrum.

Mechanisms by which SGLT2i reduce progression to diabetes or intensification of GLT in HF are uncertain, but likely multifactorial. Foremost, the glucosuric effects of SGLT2i may help to maintain or restore normoglycaemia among individuals at increased risk for incident diabetes, such as those with pre-diabetes. Although competed trials, including DAPA-HF and DAPA-CKD, have not shown significant effects of SGLT2i on HbA1c in the absence of diabetes,^8^ this may, in part, also reflect efforts to improve glycaemic control after identification of abnormal HbA1c levels, which were unblinded. Further, SGLT2i are known to promote reductions in weight and visceral/ectopic adiposity,^10,23^ the former of which accounted for the majority of the favourable effects of metformin on incident diabetes in the Diabetes Prevention Program.^29^ Of note, it has been suggested that these findings and others from dedicated diabetes prevention trials may be due to transient glycaemic effects alone (i.e. ‘masking’ of underlying diabetes) rather than modification of the underlying pathobiology.^29,30^ However, the need for GLT as shown in this analysis (as a marker of clinically significant diabetes), combined with other established benefits of SGLT2i on metabolism (which may improve insulin sensitivity directly), health status (which may improve insulin sensitivity indirectly through increased physical activity), and, potentially, pancreatic β-cell function,^31^ makes a masking effect less likely. Overall, there is need for further research efforts evaluating mechanisms and predictors of incident diabetes in HF.

Taken together, because even optimal treatment may not normalize additive risk, need for disease-modifying pharmacotherapy, or cost of care once diabetes is established among individuals with HF, upstream preventive actions are needed. In addition to established benefits of SGLT2i on death, disability, and cardiovascular-kidney-metabolic health,^24,32^ this analysis highlights the potential of SGLT2i, as part of comprehensive strategies targeting modifiable risk factors (e.g. excess adiposity, physical inactivity, and adverse dietary habits), to prevent or delay diabetes and related sequelae among individuals with cardiovascular or kidney disease.

### Limitations

Several limitations of this analysis should be highlighted. First, as serial measurement of HbA1c was not available in DELIVER, our definition of incident diabetes relied on new initiation of GLT. Although this led to underestimation of the true incidence of diabetes (and, possibly, the absolute effects of SGLT2i on new-onset diabetes), the relative treatment effect size observed in this analysis was highly comparable to those observed in prior studies in which serial HbA1c was available,^8,9^ suggesting that new GLT initiation is a meaningful surrogate for new diabetes in this population. This approach of centring the endpoint definition around requiring new treatment (rather than including changes in laboratory measures alone) may additionally be more clinically relevant and digestible for patients and providers. Second, GLT may have been initiated for indications other than diabetes, such as pre-diabetes or weight management. However, GLT initiation for these reasons was likely rare, and avoidance of additional pharmacotherapy irrespective of the indication may be important in this population adversely impacted by a high prevalence of polypharmacy.^33^ Further, similar reduction in new-onset diabetes was observed even after exclusion of biguanides and glucagon-like peptide-1 receptor agonists, GLTs most likely to be initiated for pre-diabetes and/or weight management. Third, we did not consider duration of GLT use, and some initiation might have been transient. Fourth, we were unable to ascertain the type of new-onset diabetes during trial follow-up. However, in keeping with epidemiological analyses involving this age group,^34^ we suspect that the vast majority of new-onset diabetes was type 2 diabetes. Fifth, as these trials were not explicitly designed to evaluate the effect of SGLT2i on incident diabetes, power to examine treatment-by-subgroup interactions was limited. Sixth, we were unable to harmonize definitions of new-onset diabetes across trials; use of varying definitions for new-onset diabetes may have introduced bias. Seventh, serial fasting glucose measurements, oral glucose tolerance testing, and study drug washouts were not performed in the included trials. Finally, these findings from clinical trials may not be generalizable to all populations.

## Conclusions

SGLT2i reduced incident diabetes necessitating the initiation of GLT in patients with HF across the LVEF spectrum, without excess risk of major hypoglycaemia. In a comprehensive meta-analysis of seven trials of patients with cardiovascular or kidney diseases, we estimate that SGLT2i reduce risk of new-onset diabetes by 26%. These findings further emphasize the role of SGLT2i as a core component of comprehensive strategies to improve cardiovascular-kidney-metabolic health.

## Supplementary Material

ehae780_Supplementary_Data

## Declarations

### Data Availability

Data underlying the findings described in this manuscript may be obtained in accordance with AstraZeneca’s data sharing policy described at https://astrazenecagrouptrials.pharmacm.com/ST/Submission/Disclosure.  Data for studies directly listed on Vivli can be requested through Vivli at www.vivli.org. Data for studies not listed on Vivli could be requested through Vivli at https://vivli.org/members/enquiries-about-studies-not-listed-on-the-vivli-platform/. AstraZeneca Vivli member page is also available outlining further details: https://vivli.org/ourmember/astrazeneca/.

### Pre-registered Clinical Trial Number

DAPA-HF: NCT03036124. DELIVER: NCT03619213.
