# Supplementary material for: Sodium-glucose co-transporter 2 inhibitors and new-onset diabetes in cardiovascular or kidney disease
Source: Eur Heart J. 2024 Nov 21;46(14):1321–31. doi: 10.1093/eurheartj/ehae780 (PMC11973562; doi:10.1093/eurheartj/ehae780)
Supplement: ehae780_Supplementary_Data [file ehae780_supplementary_data.docx]

**Sodium-Glucose Co-Transporter 2 Inhibitors and New-Onset Diabetes in Cardiovascular or Kidney Disease**

Ostrominski JW et al.

**Table of Contents**

1. Supplementary Methods: Search Strategy 2
2. Supplementary Table 1: Trial Characteristics and New-Onset Diabetes Definitions 3
3. Supplementary Table 2: Key Baseline Characteristics of Included Cardiovascular and Kidney Trials 4
4. Supplementary Figure 1: PRISMA Flow Diagram for Study Selection 5
5. Supplementary Figure 2: Cumulative Incidence of New-Onset Diabetes Requiring Glucose-Lowering 6

Therapy in DAPA-HF and DELIVER

1. Supplementary Figure 3: Treatment Effects of Dapagliflozin versus Placebo on New-Onset Diabetes by 7 Continuous Baseline Body Mass Index and Glycated Hemoglobin in Pooled Analysis of DAPA-HF and DELIVER

**Supplementary Methods: Preregistered Search Strategy for Large-Scale Randomized Clinical Outcomes Trials Evaluating the Effect of SGLT2i on New-Onset Diabetes in Cardiovascular or Kidney Disease**

The following string was used in the PubMed/MEDLINE prespecified search query to identify potential studies to be included in the meta-analysis:

PubMed Query: Search: (("randomized controlled trial"[Title/Abstract] OR "clinical trial"[Title/Abstract] OR "placebo"[Title/Abstract] OR "placebo-controlled"[Title/Abstract]) AND ("empagliflozin"[Title/Abstract] OR "dapagliflozin"[Title/Abstract] OR "ertugliflozin"[Title/Abstract] OR "sotagliflozin"[Title/Abstract] OR "canagliflozin"[Title/Abstract] OR "bexagliflozin"[Title/Abstract]) AND ("diabetes"[Title/Abstract]) AND ("heart failure"[Title/Abstract] OR "cardiovascular disease"[Title/Abstract] OR "kidney disease"[Title/Abstract] OR "myocardial infarction"[Title/Abstract]) AND "humans"[MeSH Terms]) NOT ("acute heart failure"[Title/Abstract] OR "acute decompensated"[Title/Abstract] OR "acute kidney"[Title/Abstract] OR "review"[Title/Abstract] OR "meta-analysis"[Title/Abstract])

Articles resulting from these searches and relevant references cited in those articles were reviewed. Articles published in a language other than English were excluded.

**Supplementary Table 1: Trial Characteristics and New-Onset Diabetes Definitions**

| **Trial** | **ClinicalTrials.gov Identifier** | **Enrollment Period** | **SGLT2i** | **Key Inclusion Criteria** | **Post-Baseline HbA_1c_ Collection** | **New-Onset Diabetes Definition** |
| --- | --- | --- | --- | --- | --- | --- |
| **Cardiovascular Trials** | | | | | | |
| DAPA-HF | NCT03036124 | Feb 2017 to Aug 2018 | Dapagliflozin | 1. HF and LVEF ≤40% 2. NYHA II-IV 3. With or without T2D | Yes | Clinical diagnosis requiring glucose-lowering therapy or HbA_1c_ ≥6.5% on 2 consecutive follow-up visits* |
| DAPA-MI | NCT04564742 | Dec 2020 to Mar 2023 | Dapagliflozin | 1. Acute MI 2. Impaired LV systolic function 3. No prior HF or T2D | Yes | Investigator diagnosis of diabetes requiring glucose-lowering therapy or HbA_1c_ ≥6.5% at ≥2 time points |
| DELIVER | NCT03619213 | Aug 2018 to Dec 2020 | Dapagliflozin | 1. HF and LVEF >40% 2. NYHA II-IV 3. With or without T2D | No | New initiation of glucose-lowering therapy |
| EMPEROR-Preserved | NCT03057951 | Mar 2017 to Apr 2020 | Empagliflozin | 1. HF and LVEF >40% 2. NYHA II-IV 3. With or without T2D | Yes | Among participants with prediabetes, an investigator diagnosis of diabetes or ≥1 HbA_1c_ value ≥6.5% |
| EMPEROR-Reduced | NCT03057977 | Apr 2017 to Nov 2019 | Empagliflozin | 1. HF and LVEF ≤40% 2. NYHA II-IV 3. With or without T2D | Yes | Among participants with prediabetes, an investigator diagnosis of diabetes or ≥1 HbA_1c_ value ≥6.5% |
| **Kidney Trials** | | | | | | |
| DAPA-CKD | NCT03036150 | Feb 2017 to Oct 2018 | Dapagliflozin | 1. CKD (eGFR 25-75 mL/min/1.73 m^2^ and UACR 200-5000 mg/g) 2. With or without T2D | Yes | ≥1 HbA_1c_ value ≥6.5% |
| EMPA-KIDNEY | NCT03594110 | Feb 2019 to Apr 2021 | Empagliflozin | 1. CKD (eGFR 20 to <45 mL/min/1.73 m^2^ or eGFR 45 to <90 mL/min/1.73 m^2^ with UACR ≥200 mg/g) 2. With or without T2D | Yes | Clinical diagnosis of diabetes, initiation of glucose-lowering therapy, or ≥1 HbA_1c_ value ≥6.5% |

*: In this meta-analysis, a new-onset diabetes definition of new initiation of glucose-lowering therapy was employed for harmonization with DELIVER

Abbreviations: CKD = chronic kidney disease; DAPA-CKD = Dapagliflozin and Prevention of Adverse Outcomes in Chronic Kidney Disease; DAPA-HF = Dapagliflozin and Prevention of Adverse Outcomes in Heart Failure; DAPA-MI = Dapagliflozin in Patients with Myocardial Infarction; DELIVER = Dapagliflozin Evaluation to Improve the Lives of Patients with Preserved Ejection Fraction Heart Failure; eGFR = estimated glomerular filtration rate; EMPA-KIDNEY = Study of Heart and Kidney Protection with Empagliflozin; EMPEROR-Preserved = Empagliflozin Outcome Trial in Patients with Chronic Heart Failure with Preserved Ejection Fraction; EMPEROR-Reduced = Empagliflozin Outcome Trial in Patients with Chronic Heart Failure and a Reduced Ejection Fraction; HbA_1c_ = glycated hemoglobin; HF = heart failure; LV = left ventricle/ventricular; LVEF = LV ejection fraction; MI = myocardial infarction; NYHA = New York Heart Association; SGLT2i = sodium-glucose co-transporter 2 inhibitor; T2D = type 2 diabetes; UACR = urine albumin-to-creatinine ratio

**Supplementary Table 2: Key Baseline Characteristics of Included Cardiovascular and Kidney Trials**

|  | **Cardiovascular Trials** | | | | | | **Kidney Trials** | |
| --- | --- | --- | --- | --- | --- | --- | --- | --- |
| **Characteristic** | **DAPA-HF**  **(n=4744)** | **DAPA-MI**  **(n=4017)** | **DELIVER**  **(n=6263)** | **EMPEROR-Preserved**  **(n=5988)** | **EMPEROR-Reduced**  **(n=3730)** | **DAPA-CKD**  **(n=4304)** | | **EMPA-KIDNEY**  **(n=6609)** |
| Age, y | 66 | 63 | 72 | 72 | 67 | 62 | | 64 |
| Women, % | 23 | 20 | 44 | 45 | 24 | 33 | | 33 |
| History of heart failure, % | 100 | 0 | 100 | 100 | 100 | 11 | | 10 |
| History of hypertension, % | 74 | 37 | 89 | 90 | 72 | 96 | | -- |
| History of cardiovascular disease, % | -- | -- | -- | -- | -- | 37 | | 27 |
| History of myocardial infarction, % | 44 | 9 | 26 | 29 | -- | 9 | | -- |
| History of stroke, % | 10 | 2 | 9 (stroke/TIA) | 10 | -- | 7 | | -- |
| History of type 2 diabetes, % | 42 | 0 | 45 | 49 | 50 | 68 | | 44 |
| Body mass index, kg/m^2^ | 28 | -- | 30 | 30 | 28 | 30 | | 30 |
| Baseline LVEF, mean, % | 31 | -- | 54 | 54 | 27 | -- | | -- |
| NT-proBNP, median, pg/mL | 1437 | -- | 1011 | 974 | 1907^*^ | -- | | 190 |
| eGFR, mean, mL/min/1.73 m^2^ | 66 | 83 | 61 | 61 | 62 | 43 | | 38 |
| β-blocker, % | 96 | 90 | 76 | 86 | 95 | 39 | | 42 |
| ACEi, % | 56 | -- | 33 | 40 | -- | 32 | | -- |
| ARB, % | 28 | 23 | 34 | 39 | -- | 67 | | -- |
| ARNI, % | 11 | -- | 4 | 2 | 20 | 0.1 | | -- |
| RAS inhibitor, % | 94^**^ | 92 | -- | -- | 89^**^ | -- | | 85 |
| MRA, % | 71 | -- | 39 | 37 | 71 | 5 | | 7 |
| Diuretic, % | 93 | -- | Loop: 72 | 86 | -- | Loop: 25  Thiazide: 21 | | Loop: 26  Thiazide: 16 |

Values are %, mean, or median (as reported).

^*^: Approximate value; average of medians reported by treatment arm.

^**^: Including ARNI.

Abbreviations: ACEi = angiotensin-converting enzyme inhibitor; ARB = angiotensin receptor blocker; ARNI = angiotensin receptor-neprilysin inhibitor; DAPA-CKD = Dapagliflozin and Prevention of Adverse Outcomes in Chronic Kidney Disease; DAPA-HF = Dapagliflozin and Prevention of Adverse Outcomes in Heart Failure; DAPA-MI = Dapagliflozin in Patients with Myocardial Infarction; DELIVER = Dapagliflozin Evaluation to Improve the Lives of Patients with Preserved Ejection Fraction Heart Failure; eGFR = estimated glomerular filtration rate; EMPA-KIDNEY = Study of Heart and Kidney Protection with Empagliflozin; EMPEROR-Preserved = Empagliflozin Outcome Trial in Patients with Chronic Heart Failure with Preserved Ejection Fraction; EMPEROR-Reduced = Empagliflozin Outcome Trial in Patients with Chronic Heart Failure and a Reduced Ejection Fraction; LVEF = LV ejection fraction; MRA = mineralocorticoid receptor antagonist; NT-proBNP = N-terminal prohormone of B-type natriuretic peptide

**Supplementary Figure 1: PRISMA Flow Diagram**

**Supplementary Figure 2: Cumulative Incidence of New-Onset Diabetes Requiring Glucose-Lowering Therapy in DAPA-HF and DELIVER**


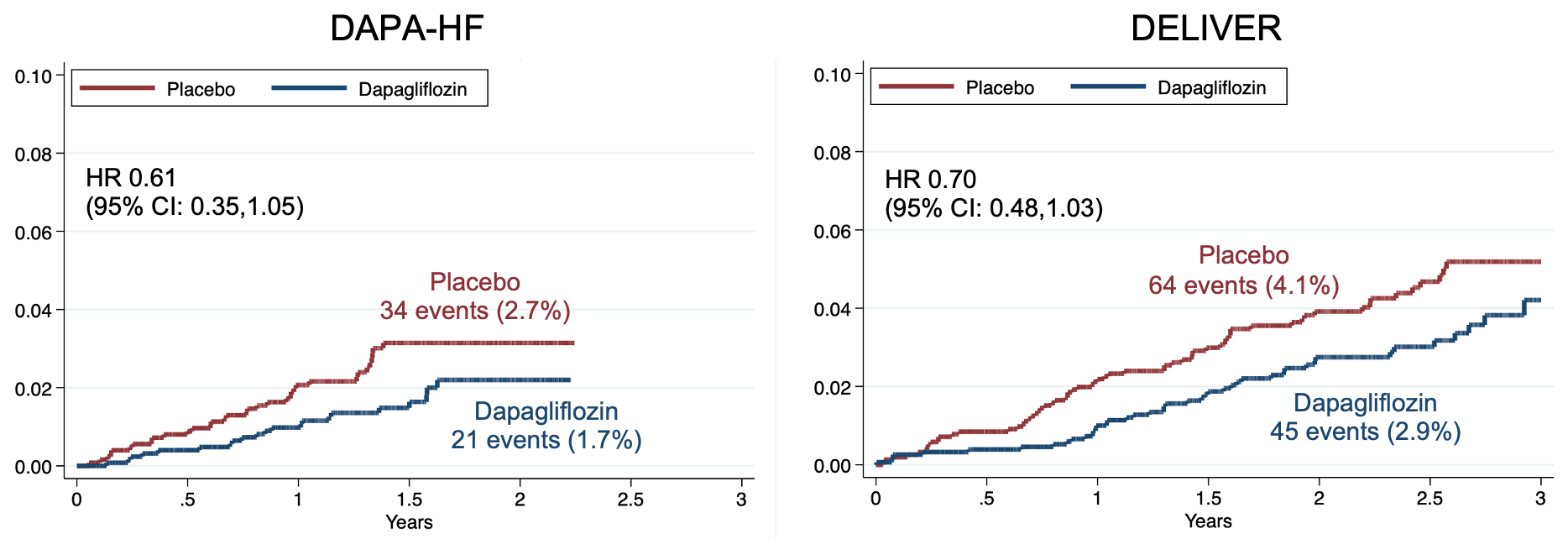


Abbreviations: DAPA-HF = Dapagliflozin and Prevention of Adverse Outcomes in Heart Failure; DELIVER = Dapagliflozin Evaluation to Improve the Lives of Patients with Preserved Ejection Fraction Heart Failure; HR = hazard ratio.

**Supplementary Figure 3: Treatment Effects of Dapagliflozin versus Placebo on New-Onset Diabetes by Continuous Baseline Body Mass Index and Glycated Hemoglobin in Pooled Analysis of DAPA-HF and DELIVER**

**
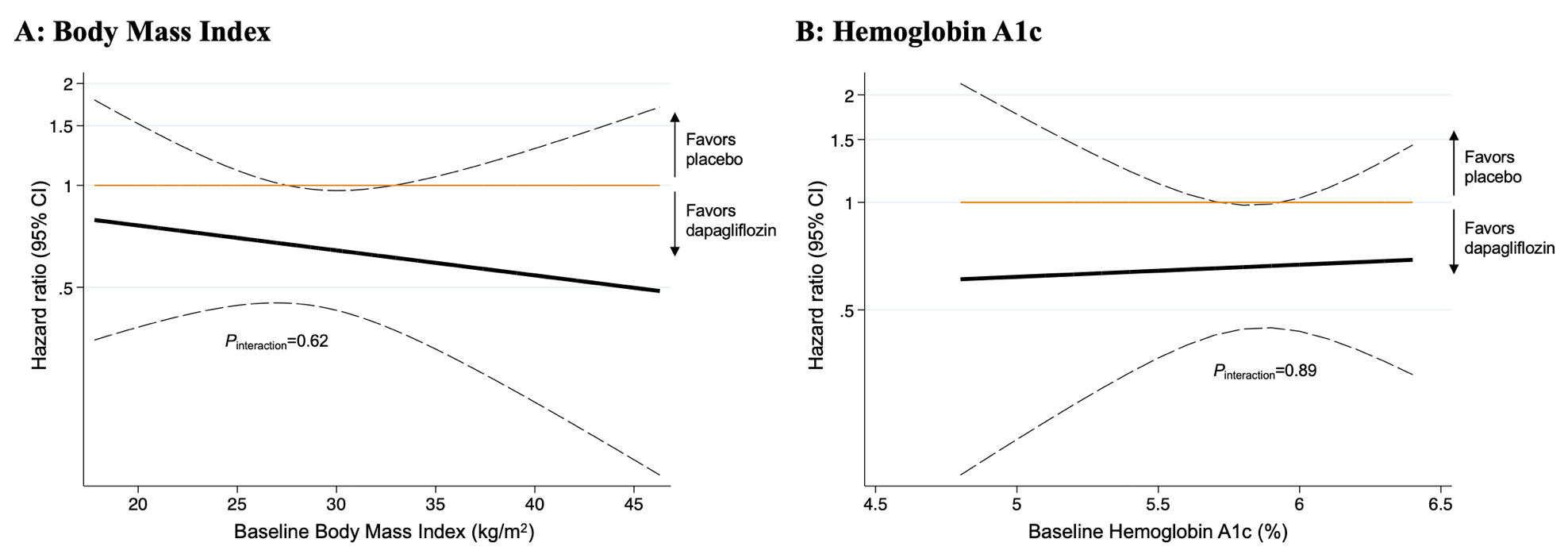
**

Estimated hazard ratios (solid black line) and 95% confidence intervals (dotted black lines) obtained from Cox models with baseline continuous body mass index (A) and glycated hemoglobin (B) expressed via a linear model.

Abbreviations: DAPA-HF = Dapagliflozin and Prevention of Adverse Outcomes in Heart Failure; DELIVER = Dapagliflozin Evaluation to Improve the Lives of Patients with Preserved Ejection Fraction Heart Failure.
